# Impact of Longitudinal Changes in Dynamic Lower-Limb Function on Care-Needs Certification: A 4-Year Prospective Study of Older Women Attending Community Gathering Places (*Kayoi-no-ba*)

**DOI:** 10.3390/healthcare14183009

**Published:** 2026-09-14

**Authors:** Tadayuki Iida, Wakako Tsumiyama, Natsumi Murao, Yuta Sato, Hiroka Ito, Kazumi Kayano

**Affiliations:** 1 Physical Therapy, Department of Health and Welfare, Faculty of Health and Welfare, Prefectural University of Hiroshima, 1-1 Gakuencho, Mihara 723-0053, Hiroshima, Japan; tsumiyama@pu-hiroshima.ac.jp (W.T.); y-satou@pu-hiroshima.ac.jp (Y.S.); 2 Citizen Welfare Department, Takehara City Office, 5-6-28 Chuo, Takehara 725-8666, Hiroshima, Japan; shimin@city.takehara.lg.jp (N.M.); kaigo@city.takehara.lg.jp (H.I.); kenkou@city.takehara.lg.jp (K.K.)

**Keywords:** physical performance measures (PPMs), Timed Up and Go (TUG), 30-second chair stand test (CS-30), care-needs certification, Kayoi-no-ba

## Abstract

**Background**: Japan’s rapidly aging society faces a critical challenge in sustaining the public long-term care insurance (LTCI) system. Community gathering places (Kayoi-no-ba) have been promoted for preventive care, but the predictive value of longitudinal changes in physical performance measures (PPMs) within these settings is not fully understood. **Objectives**: Our objective was to clarify the association between baseline PPMs and their longitudinal change over four years with subsequent care-needs certification among community-dwelling older women. **Methods**: We conducted a 4-year prospective study of independent older women (*n* = 223–226), assessing handgrip strength (HGS), Timed Up and Go (TUG), the 30-second chair stand test (CS-30), and the one-leg standing test (OLST). Analysis 1 examined baseline risk; Analysis 2 tracked functional transition (maintenance vs. decline) among those at “no risk” at baseline. **Results**: Baseline PPM status was not significantly associated with future care-needs certification after adjusting for age and BMI. A longitudinal decline in TUG and CS-30 was associated with a significantly higher risk of certification (TUG: Hazard Ratio [HR] = 17.34, *p* < 0.001; CS-30: HR = 7.087, *p* = 0.002), which remained significant after Benjamini–Hochberg false discovery rate correction for multiple comparisons. However, a sensitivity analysis excluding participants certified before their 2023 re-measurement showed that the CS-30 association was no longer supported, while TUG remained nominally significant but with an extremely wide confidence interval (HR = 18.99, 95% CI 1.155–312.390, *p* = 0.039) reflecting very few events. A complementary continuous-change analysis corroborated this pattern (TUG: HR = 2.34 per 1-SD worsening, 95% CI 1.64–3.33, *p* < 0.001); no association was observed for HGS, CS-30, or OLST in this or the primary analyses. **Conclusions**: A longitudinal decline in TUG performance may be associated with increased care-needs certification risk among older women, but this finding is sensitive to the temporal ordering of measurement and certification and requires confirmation in larger, prospective studies. The CS-30 finding did not withstand sensitivity analysis and should not be treated as a robust predictor.

## 1. Introduction

Japan is the world’s most rapidly aging society, and effective community-based preventive care is essential to extend healthy life expectancy [1]. The increase in long-term care benefit expenditures has become a critical issue for the financial sustainability of the country [2]. Estimates suggest that population aging and advances in medical technologies could drive medical and long-term care spending to ¥83 trillion by 2040, a 1.5-fold increase over the 2020 figure [3]. Consistent with the convention used in official Japanese government social security projections of this type, this estimate is expressed in nominal terms rather than adjusted for future inflation; the real-terms increase may therefore be more modest than the nominal figure suggests. Consequently, “preventive care” measures aimed at preventing older adults from requiring long-term care and extending healthy life expectancy have been the core of policy since the introduction of the public long-term care insurance (LTCI) system in 2000. Since the promotion of the Community-Based Integrated Care System by the Ministry of Health, Labour and Welfare in 2014, “General Projects for Care Prevention and Daily Life Support” led by municipalities have been developed, within which the expansion of resident-operated “community gathering places (*Kayoi-no-ba*)” has been promoted [4]. *Kayoi-no-ba* serve as a unique model that simultaneously promotes social participation and physical activity [5]. Regular participation in these programs has been shown to reduce frailty prevalence by approximately 3.9 percentage points [6] and lower the risk of functional disability by nearly 50% [7].

Physical performance measures (PPMs), such as handgrip strength (HGS), Timed Up and Go (TUG), 30-second chair stand test (CS-30), and one-leg standing test (OLST), are widely used for clinical assessment. Of these, HGS is a static (isometric) strength measure, whereas TUG, CS-30, and OLST are dynamic measures of mobility, lower-limb power, and balance under movement. Collectively, these measures reflect mobility, lower limb muscle strength, and balance and are strongly associated with falls and physical disability. In particular, HGS is a simple and reliable proxy for whole-body muscle strength and a known predictor of mortality, morbidity, and future risk of care needs certification [8].

While these epidemiological associations are well documented in the general community-dwelling elderly population, longitudinal studies specifically tracking PPMs among active participants in community-based gathering places (*Kayoi-no-ba*) are limited. Most existing studies still rely on cross-sectional frameworks or focus primarily on general community dwellers rather than those actively participating in specific social hubs [9,10]. Furthermore, traditional frailty markers, such as HGS, often exhibit a “ceiling effect” when applied to relatively healthy, socially active older adults, potentially failing to capture the earliest, subtler signs of functional decline [11].

Therefore, rather than relying on cross-sectional data or static single-point screenings, this study emphasizes the importance of tracking longitudinal “trajectories” of physical function [12]. Theoretically, this approach aligns with the Disablement Process Model, in which functional limitations represent an identifiable intermediate stage between impairment and disability rather than a mere downstream consequence of overt disease [13]. Within this framework, subtle declines in dynamic lower-limb performance may constitute a stage of “preclinical disability” that precedes, and is potentially distinguishable from, the disease-driven pathway to care-needs certification [14]. By focusing on dynamic lower-limb functions (TUG and CS-30) that are more closely linked to complex activities of daily living (ADLs) and functional independence, we aimed to clarify the distinct predictive value of functional transitions within Japan’s public long-term care insurance (LTCI) system.

Women are prone to age-related declines in muscle mass and strength (sarcopenia), with reported annual decline rates in lower-extremity physical performance of approximately 1.8–3.0% in women compared with 1.4–2.8% in men [15], making their independent living vulnerable to osteoporosis and falls [16]. In Japan, approximately 70% of those certified for care needs are women [17]. Furthermore, postmenopausal women are biologically vulnerable to skeletal muscle loss due to estrogen deficiency, which accelerates mitochondrial dysfunction and myofiber loss [18]. Therefore, examining the relationship between PPMs and care-needs certification, focusing on women, is highly significant for prioritizing preventive care policies and providing individualized support. We note that these sex-related biological considerations motivate our focus on women as a population of particular clinical concern; this study was not designed as a sex-comparative analysis, and no men were included in the analytic sample (see Section 2.1). While isometric strength measures such as HGS capture gross muscle mass decay, they fail to reflect the age-related loss of neuromuscular coordination, muscle power, and dynamic balance required for complex daily tasks. In contrast, dynamic performance measures integrate velocity and multi-joint coordination, making their longitudinal trajectories more sensitive to early stage mobility disability in high-functioning cohorts [19,20].

The present study aimed to clarify the association between PPMs (HGS, TUG, CS-30, and OLST) at baseline (2019) and care-needs certification over a 4-year period (2020–2023) among community-dwelling older adults who were not certified at baseline. Furthermore, among those whose PPMs were above the reference value at baseline, we aimed to compare those who fell below the reference value after 4 years with those who maintained it, examining the association with care-needs certification.

## 2. Materials and Methods

### 2.1. Participants and Ethical Considerations

**Figure 1:**
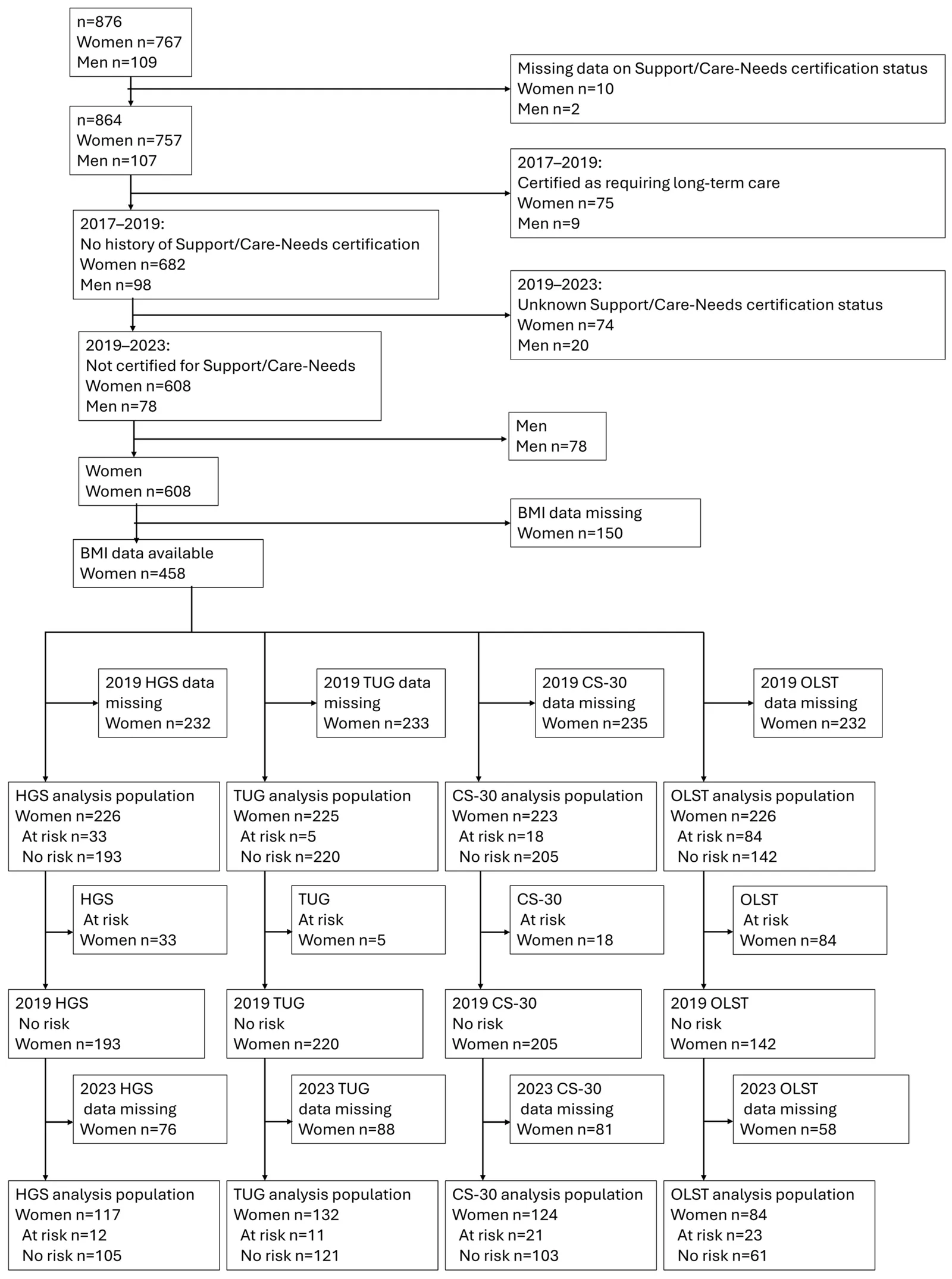
Flowchart of the analyzed participants. HGS, handgrip strength; TUG, Timed Up and Go test; CS-30, 30-second chair stand test; OLST, one-leg standing test.

This study targeted community-dwelling older adults participating in community gathering places (*Kayoi-no-ba*) in Takehara City. Participants were provided with written documents outlining the study’s objectives and procedures, and written informed consent was obtained after a verbal explanation. Between 2017 and 2023, 876 individuals (767 women and 109 men) underwent PPMs (HGS, TUG, CS-30, and OLST) at least once. Linkable anonymized data regarding Support and Care-needs certification from 2017 to 2023 were provided by Takehara City with official consent. Twelve individuals (10 women and 2 men) for whom certification data were unavailable were excluded. With 2019 as the baseline and 2023 as the follow-up period, 686 individuals (608 women and 78 men) who were not certified at baseline and whose status could be tracked were identified. Men (*n* = 78) were excluded from the analysis. Furthermore, individuals missing baseline age or BMI data used as confounding adjustment factors (*n* = 150) were excluded. After excluding those with missing baseline PPM values, the participants for Analysis 1 were as follows: HGS (*n* = 226), TUG (*n* = 225), CS-30 (*n* = 223), and OLST (*n* = 226) measurements. In Analysis 1, participants were classified into “at-risk” and “no-risk” groups based on baseline PPM cutoff values. Analysis 2 targeted individuals from the baseline “no-risk” group who also underwent re-measurement in 2023 (HGS, *n* = 117; TUG, *n* = 132, CS-30 *n* = 124; OLST, *n* = 84). The selection process is illustrated in Figure 1. The present study was conducted in accordance with the ethical principles of the Declaration of Helsinki and was approved by the Ethics Committee of the Prefectural University of Hiroshima (Approval No. 24MH013, date of approval: 28 June 2024).

### 2.2. Measurements

From 2017 to 2023, height (cm) and weight (kg) were measured annually for community-dwelling older adults participating in *Kayoi-no-ba* in Takehara City, and BMI was calculated from these values. Four items were measured as PPMs: HGS, TUG, CS-30, and OLST. These four PPMs were selected to capture both static strength (HGS) and dynamic functions (TUG test, CS-30), which are critical for maintaining independence in community-dwelling older adults. Some participants were unable to perform certain tests owing to physical constraints. HGS was measured using a digital dynamometer (Grip-D TKK-5401 Smedley; Takei Scientific Instruments, Niigata, Japan), and a stopwatch (SVAJ003; Seiko Watch Corp., Tokyo, Japan) was used for the timed tests. Consistent with standard clinical dynamometry practice and with the cited reference values [21], HGS is reported in kilograms of force (kgf, denoted kg throughout) rather than the SI-derived unit of force (newtons). For HGS, two trials were performed and the maximum value was recorded. For TUG, two trials were performed and the faster (shorter) time was recorded. CS-30 was assessed with a single trial. For OLST, two trials were performed and the longer time was recorded, except that only one trial was conducted if a participant reached the 60-s ceiling on the first attempt. Measurements were performed by physical therapists and public health nurses who supported *Kayoi-no-ba*. All assessors were trained on a standardized measurement manual prior to data collection; however, formal inter-rater reliability (e.g., intraclass correlation coefficients) was not assessed. At each *Kayoi-no-ba* site, the order in which the four PPMs were administered was randomized by the investigators; no fixed order was used.

Reference values were used to define “at-risk” status as follows:

- HGS (possible sarcopenia): <18 kg for women based on the Asian Working Group for Sarcopenia (AWGS 2019) criteria [21].
- TUG and OLST (locomotor instability): According to the diagnostic criteria for Musculoskeletal Ambulation Disability Symptom Complex (MADS) defined by the Japanese Orthopaedic Association, impaired mobility is identified using physical performance tests. A TUG time of ≥11 s and/or an OLST time of <15 s were considered indicative of an increased risk of falls [22].
- CS-30: Based on the report by Sawada et al., ≤15 repetitions were defined as the risk threshold related to sarcopenia [23].

### 2.3. Care-Needs Certification

Care-needs certification under the public long-term care insurance (LTCI) system in Japan is determined using nationally standardized criteria based on an individual’s physical status and cognitive function. Certification levels are categorized into seven stages: “Support Level 1–2,” for individuals requiring partial assistance with daily activities, and “Care Level 1–5,” for those requiring assistance with activities of daily living (ADL). The required amount of care increases proportionally to the certification level. The certification process consists of a primary computer-aided assessment based on a survey of mental and physical conditions (certification survey) conducted by certification investigators and a primary care physician’s opinion (Doctor’s Opinion Paper). The primary assessment is followed by a secondary assessment by the Health Care and Social Welfare Certification Committee, composed of academic experts in health, medicine, and welfare, to determine the final classification. In this study, the primary endpoint (event occurrence) was defined as the receipt of any of these new certifications for the first time during the follow-up period [24].

### 2.4. Statistical Analysis

The primary outcome was the timing of new care-needs certification. Because the event of interest was administrative long-term care certification rather than a disease occurrence or recurrence, the outcome period is referred to throughout as “time to care-needs certification” rather than “disease-free survival.” The follow-up period spanned from the 2019 baseline to 2023. Total person-time of observation was approximately 852–862 person-years across the four Analysis 1 cohorts and approximately 332–506 person-years across the four Analysis 2 cohorts, depending on the measure; exact certification event counts, proportions, and median follow-up by subgroup are reported in the footnotes to the corresponding tables below.

The minimum sample size was initially evaluated using G*Power (version 3.1) [25] based on the independent *t*-test logic using physical performance thresholds reported by Tanimoto et al. [17]. Considering their findings regarding TUG scores between individuals with and without possible sarcopenia (M_1 = 8.4, SD_1 = 3.9 vs. M_2 = 5.9, SD_2 = 1.1), a minimum sample size of approximately 21 participants per group was estimated to detect basic functional differences (two-sided α = 0.05, power = 80%). A two-sided test was adopted for this a priori calculation because published evidence indicates that the direction of the association between sarcopenia status and physical performance measures such as TUG is not always consistent and can be confounded by factors such as body height [26]; a two-sided test therefore avoids assuming a specific direction of effect a priori.

However, because the primary outcome of this study was evaluated using multivariate Cox proportional hazards regression models, we further justified the sample size based on the Events per Variable (EPV) criteria proposed by Peduzzi et al. [27] to ensure model stability. For Analyses 1 and 2, the number of observed care-needs certification events satisfied the standard statistical requirements for stable hazard ratio estimation in the CS-30 and OLST models. In contrast, for models in which the specific follow-up sample size or event rate was insufficient (e.g., baseline TUG, and 2023 follow-up HGS and TUG), the statistical power was limited, and these specific hazard ratios were strictly interpreted as exploratory findings, as detailed in the study limitations.

In Analysis 1, the participants were stratified according to their baseline risk status. The association with care-needs certification was examined using Cox proportional hazards regression models, with age and BMI as covariates. In Analysis 2, we focused on those in the baseline “no risk” group who underwent physical performance testing in 2023. They were categorized by their 2023 status to evaluate the association between the 4-year functional transition and subsequent certification using identical models. Statistical analyses were performed using SPSS version 27.0 (IBM Japan, Tokyo, Japan), with *p* < 0.05 indicating statistical significance.

## 3. Results

The basic characteristics of the study participants are presented in Table 1 and Table 2. Table 3 presents the results of the Cox proportional hazards regression analysis, stratified by risk status at baseline (Analysis 1). No significant associations were observed between baseline HGS, TUG, CS-30, or OLST and care needs certification after adjusting for covariates. To address the possibility that the exclusion of 150 women with missing baseline age or BMI introduced selection bias into Analysis 1, we additionally conducted a sensitivity analysis using multiple imputation by chained equations (MICE; 5 imputations) to recover these participants (Supplementary Table S1). The pooled estimates from the imputed datasets were consistent with the complete-case results presented in Table 3, and did not alter the overall conclusions of Analysis 1 (see Limitations Section). We also considered that participants who were unable to complete a given physical performance test at baseline (HGS, *n* = 232; TUG, *n* = 233; CS-30, *n* = 235; OLST, *n* = 232, among the 458 women with baseline age and BMI available) were excluded from Table 3, which could underestimate risk if these participants were disproportionately frail. In a worst-case sensitivity analysis, we assigned all such participants to the “at-risk” category for the corresponding measure (Supplementary Table S2). The adjusted (Model 1) associations remained non-significant for all four measures under this conservative assumption, consistent with the complete-case findings in Table 3. Table 4 presents the results of the Cox proportional hazards regression analysis (Analysis 2), which targeted individuals who were not at risk at baseline and stratified them based on their physical performance in 2023. Significant associations with care-needs certification were observed for TUG and CS-30 (TUG: Hazard Ratio [HR] = 17.34, *p* < 0.001; CS-30: HR = 7.087, *p* = 0.002). No significant associations were identified between HGS and OLST. For HGS, the crude and adjusted hazard ratios were not meaningfully estimable (crude HR ≈ 0.000, 95% CI: 0–Inf; Model 1 HR = 0.000, 95% CI: 0–Inf), reflecting monotone likelihood (quasi-complete separation) driven by the near-absence of certification events within the small at-risk subgroup (*n* = 12) [28]. This near-zero value should therefore not be interpreted as evidence of an association, in either direction, between HGS and care-needs certification.

Because Analysis 2 classified participants according to their 2023 physical performance status while certification events could occur at any point during the 2019–2023 follow-up period, we conducted a post hoc sensitivity analysis excluding participants who had already received Support/Care-Needs certification before their 2023 re-measurement (Table 5). After this exclusion, the CS-30 association was no longer statistically significant in either the crude or adjusted model (Model 1: *p* = 0.955), and the hazard ratio could not be reliably estimated, indicating that the originally observed association was substantially influenced by participants whose certification preceded their final performance measurement. The TUG association remained nominally significant in both models (Model 1: HR = 18.99, 95% CI 1.155–312.390, *p* = 0.039), but the extremely wide confidence interval, driven by very few remaining events, indicates that this finding should be interpreted as exploratory rather than confirmatory. Estimates for HGS and OLST likewise could not be reliably computed in the sensitivity analysis. As a complementary analysis, we also modeled the standardized 4-year change in each measure as a continuous covariate rather than a binary risk category (Table 6). This continuous-change analysis corroborated the pattern seen above: the per-1-SD worsening in TUG was associated with a significantly higher hazard of certification in both the crude and adjusted models (Model 1: HR = 2.34, 95% CI 1.64–3.33, *p* < 0.001), whereas the corresponding associations for CS-30 (Model 1: *p* = 0.126), HGS (Model 1: *p* = 0.325), and OLST (Model 1: *p* = 0.971) were not statistically significant. This convergence across two independent analytic approaches—categorical threshold-crossing (Table 4) and continuous standardized change (Table 6)—strengthens confidence that the TUG association is the most robust finding of this study, while the CS-30 association appears comparatively fragile.

## 4. Discussion

The association between physical functional decline and care-needs certification has been clarified in numerous studies. Rantanen et al., in a study of older women with disabilities, concluded that handgrip strength (HGS) is a powerful indicator for predicting mortality risk [8]. Makizako et al. conducted a study on independent older adults aged 65 years and older and reported that performance in the Timed Up and Go (TUG) test and the five-times sit-to-stand test serves as an indicator for predicting future transition to a state requiring long-term care [29]. While existing research has predominantly targeted disabled individuals or the general community-dwelling population, longitudinal data on consistent participants in community gathering places (*Kayoi-no-ba*) remain sparse. Given the limited longitudinal research on functionally independent community-dwelling older women, this study holds significant value in examining functional changes at the pre-frailty stage in relation to care-needs certification [30]. In particular, establishing benchmark values for the initial TUG and CS-30 assessments is crucial for setting personalized goals, which enhances self-efficacy and encourages sustained participation in the programs. Our results support this hypothesis.

The most notable finding of this study is that longitudinal functional decline, specifically in dynamic lower-limb measures, was associated with care-needs certification more strongly than cross-sectional status at a single baseline point, although, as detailed below, this association was not equally robust across measures once the temporal ordering of certification and measurement was accounted for. Among participants who were initially in the “no risk” group, those whose dynamic lower-limb function declined to the “at risk” category (TUG ≥ 11 s, CS-30 ≤ 15 repetitions) during the follow-up period had a higher rate of care-needs certification than those who maintained their function, consistent with a previous two-year study in which older adults with TUG times under 9 s were less likely to require support or care [29], and with Guralnik et al., who reported that poorer lower-limb function predicted higher mortality and institutionalization rates [31]. The TUG integrates walking, chair-rising, and turning ability [32]; the CS-30 correlates strongly with quadriceps strength, captures muscle power, endurance, and whole-body dynamic balance [33,34], and is more responsive to strength changes than HGS [35], and a relative decline of 28% over three years has been identified as a clinically meaningful threshold for ADL disability risk [36]. This dynamic decline may be accompanied by a vicious cycle of deconditioning, in which diminishing lower-limb power restricts life-space mobility (e.g., the frequency of outings to Kayoi-no-ba) and accelerates frailty progression [5,37]. Our findings suggest that continuous changes in dynamic lower-limb function, particularly TUG, may provide additional information beyond single-point screening; however, as our sensitivity analysis indicates, this signal was robust only for TUG, and even that association should be interpreted cautiously given its dependence on a very small number of events once reverse temporal ordering was addressed.

In contrast, no significant association was found between HGS and OLST in the two groups. Before considering possible physiological explanations, we note that statistical power was limited for both measures—particularly for HGS in Analysis 2, where only 12 of 117 participants were classified as at-risk, and to a lesser extent for OLST (23 of 84)—so a true association that this study was underpowered to detect (Type II error) cannot be excluded. The physiological interpretations below should be read with this limitation in mind. Rantanen et al. reported that, among women aged 65 and older with disabilities, those with weak HGS had higher mortality rates, suggesting that HGS may reflect disease severity, physical resilience, and nervous system efficiency [8]. While HGS is known to correlate with whole-body strength, including the lower limbs, and reflects standing balance and applied walking ability [38], some studies argue that HGS alone is insufficient for evaluating total muscular function. Furthermore, HGS dynamometry measures maximal strength and cannot capture multifaceted muscle functions such as endurance and power [39]. These considerations suggest that, while HGS is a useful indicator of one aspect of health status, it has limitations in independently predicting future care-needs certification (under the LTCI system) in a functionally independent population. This lack of association at baseline may be attributed to a “ceiling effect” in relatively high-functioning women attending *Kayoi-no-ba* (community gathering places), where single-point assessments may not capture the early stages of locomotor instability. Therefore, rehabilitation professionals should focus on the “trajectory” of performance rather than a single failed test. This ceiling effect likely explains why no significant association was found in this study, in contrast to previous studies involving more disabled cohorts [8].

The OLST is used to assess static balance [40,41] and is known to correlate with quadriceps and toe-grip strength [42]. The quadriceps are prone to early onset muscle weakness [15]. Quadriceps weakness causes a decrease in standing balance and walking ability, which can lead to falls [43]. In our study, significant associations with care-needs certification were observed for TUG and CS-30, both of which reflect lower limb dynamic capability. Additionally, age-related muscle decline is more pronounced in the lower limbs than in the upper limbs [44]. Because the participants in this study were regular attendees of *Kayoi-no-ba*, it is highly probable that only a few had advanced frailty. Consequently, the transition to care needs was likely associated with dynamic lower-limb function rather than primarily with upper-limb strength, as measured by HGS [39].

The fact that this study focused exclusively on women may have also influenced its results. Since women generally have less upper-limb muscle mass and lower absolute handgrip strength than men, HGS may be less sensitive in reflecting the risk of care-needs certification among women who maintain a certain level of activity [45,46]. Furthermore, our findings suggest that dynamic functions reflected by TUG and CS-30 were more strongly associated with care-needs risk than the OLST, a static balance indicator; however, as detailed in the Limitations, this association was not equally robust across measures once temporal ordering was accounted for, and should not be read as evidence that TUG and CS-30 predict risk more accurately in a formal, validated sense. In the stratification based on baseline physical performance measures, no significant association was observed for any of the items. Taken together, these results suggest that longitudinal functional decline, rather than cross-sectional status at a single baseline point, was associated with care-needs certification, although, as discussed above, this signal was robust only for TUG. This pattern indicates that exceeding reference values in a single measurement does not guarantee future independence; rather, it highlights the potential to identify candidates for early intervention by capturing “signs of decline” through continuous monitoring. Consequently, even among functionally independent older women, a decline in TUG was associated with a higher risk of transitioning to a state requiring care needs certification, although, as noted above, this association should be regarded as exploratory given the small number of events; the corresponding CS-30 association was not robust once temporal ordering was accounted for (see Limitations Section). Beyond the present findings, broader evidence underscores the potential downstream economic relevance of monitoring physical function: the JAGES Study, in an independent cohort, shows that cumulative LTCI costs for individuals walking < 30 min per day are approximately USD 900 higher than those walking > 60 min [47]. While our study did not directly assess economic outcomes, this supports the view that regular dynamic lower-limb function assessments may be a useful continuous monitoring indicator for community-dwelling older adults [5,29].

Capturing early signs of decline through periodic monitoring underscores the vital role of rehabilitation professionals such as physical and occupational therapists.

### Limitations

The present study had several limitations. First, our sample was limited to women from a geographically localized area (Takehara City); thus, it would be inappropriate to generalize the findings, although the participants’ heights and weights were similar to those in a Japanese national survey [48]. Second, there is a possibility that acute physical functional impairment and care-needs certification occurred simultaneously because of the onset of diseases such as stroke. To verify the factors leading to care-needs certification, it is necessary to consider various backgrounds and examine each physical function associated with the transition to a state requiring care. However, the concept of preclinical disability suggests that subclinical functional decline commonly manifests before, rather than merely alongside, overt disease onset [14], and the Disablement Process Model similarly positions functional limitation as an antecedent stage in the pathway toward disability [13]. This body of theory does not eliminate the temporal ambiguity in the present data, but it lends biological plausibility to the interpretation that the observed TUG/CS-30 decline reflects, at least in part, a genuine predictive signal rather than solely a downstream marker of incident disease. To empirically evaluate this issue, we conducted a sensitivity analysis excluding participants who had already been certified before their 2023 re-measurement (Table 5). The CS-30 association was not robust to this exclusion and should not be interpreted as a confirmed predictive relationship; the TUG association persisted nominally but was based on very few events and an extremely wide confidence interval, and should be considered hypothesis-generating rather than confirmatory. These sensitivity-analysis findings indicate that, particularly for CS-30, the originally observed association in Table 4 was substantially attributable to reverse temporal ordering rather than a genuine predictive signal. Third, regarding TUG in 2023, a significant association was observed in a group that fell below the required sample size, and the possibility that the hazard ratio was overestimated or the estimated value was unstable cannot be ruled out. Therefore, the association between TUG and care-needs certification shown in this study requires cautious interpretation, and there are limits to the generalization of the results. Future prospective studies with sufficient sample sizes are needed to verify the reproducibility and validity of these findings. However, the observed statistical significance can be positioned as an exploratory finding, suggesting that a decline in dynamic lower limb function is associated with the transition to a state requiring long-term care. Fourth, in Analysis 2, the hazard ratio for HGS exhibited monotone likelihood, yielding a near-zero point estimate with an unbounded 95% confidence interval; this reflects the sparse number of certification events within the small at-risk subgroup rather than a stable or interpretable estimate of association [28]. Fifth, this study performed multiple Cox regression models across four physical performance measures, two analyses (baseline and 4-year follow-up), and two covariate models—a total of 16 primary hazard-ratio tests. To address the resulting risk of type I error, we applied a post hoc Benjamini–Hochberg false discovery rate (FDR) correction across these 16 tests. Five associations remained statistically significant after correction (adjusted q < 0.05): baseline OLST (Table 3, crude) and both the crude and adjusted TUG and CS-30 associations in Table 4. All other associations, including those reaching only nominal (uncorrected) significance, did not survive FDR correction and should be interpreted with appropriate caution [49].

Sixth, the multivariable models were adjusted only for age and body mass index; other potentially relevant factors, including cognitive status, comorbidities, falls history, and prior hospitalization, were not available and could not be incorporated, so residual confounding cannot be excluded. In addition, the proportional-hazards assumption was formally tested for each model using a time-dependent covariate approach (covariate × ln[time] interaction terms) in IBM SPSS Statistics. This assumption was not violated (*p* > 0.05) for any model in Analysis 1 or Analysis 2, with the exception of TUG in Analysis 2, for which the test could not be reliably computed (crude model) or the model failed to converge (adjusted model)—the same sparse-data instability already noted for this subgroup (see Table 5 and the paragraph above). Regarding participants who were unable to complete a given physical performance test at baseline, a worst-case sensitivity analysis assigning these participants to the “at-risk” category did not change the non-significant Model 1 associations reported in Table 3 for any of the four measures (Supplementary Table S2), arguing against a material underestimation of risk due to their exclusion. Regarding the 150 women excluded from Analysis 1 owing to missing baseline age or BMI, a multiple imputation (MICE) sensitivity analysis produced pooled estimates consistent with the complete-case findings in Table 3 (Supplementary Table S1), suggesting that this exclusion did not materially bias the baseline results. Regarding attrition between baseline and the 2023 re-measurement in Analysis 2, participants who completed follow-up did not differ significantly from those lost to follow-up on baseline age, BMI, or baseline PPM value for any of the four measures (all *p* > 0.19), providing some reassurance against selective retention of a healthier subgroup, although unmeasured differences (e.g., in health status or motivation) cannot be excluded. Seventh, the absence of a statistically significant association for baseline physical performance measures in Analysis 1 should be interpreted as an absence of evidence rather than evidence of no association, given the imprecision of several baseline estimates (e.g., only five participants were classified as at-risk for baseline TUG); a formal comparison of the predictive performance of baseline versus longitudinal assessment (e.g., using time-dependent discrimination measures) was beyond the scope of this study but would be required to support a claim of superiority. Eighth, penalized (Firth-type) Cox regression has been proposed as a more robust approach to the sparse-data issues identified above (e.g., for HGS, and for TUG and CS-30 in the sensitivity analysis in Table 5); this could not be implemented in the present revision owing to software constraints, and represents an important direction for future analyses of this cohort, ideally combined with additional years of certification follow-up data enabling a landmark-based reanalysis (multiple imputation for the missing baseline covariates identified in the second limitation above was addressed separately; see the paragraph above and Supplementary Table S1). Ninth, the independent *t*-tests used in Table 1 and Table 2 assume approximately normal distributions; Shapiro–Wilk testing indicated that this assumption was violated for all four PPMs (*p* < 0.05). A non-parametric sensitivity check (Mann–Whitney U test) reached the same conclusion as the *t*-test for 10 of 12 baseline comparisons, but not for the BMI comparisons for HGS and TUG, where statistical significance differed between the two tests; these two comparisons should therefore be interpreted with caution, though neither is central to the study’s primary Cox regression findings.

## 5. Conclusions

In this study, individuals whose dynamic measures (TUG and CS-30) transitioned from the “no risk” group at baseline to the “at risk” group during the 4-year follow-up exhibited a higher rate of care-needs certification than those who maintained their function. However, a sensitivity analysis addressing the possibility that certification preceded, rather than followed, the assessed decline (reverse temporal ordering) indicated that this association was not supported for CS-30, and was supported for TUG only nominally, with substantial statistical uncertainty owing to a small number of events. These results suggest that longitudinal decline in TUG performance may warrant further investigation as a monitoring indicator for older women attending community-based gathering places, but do not yet provide sufficiently robust evidence to support specific clinical thresholds or a recommendation for annual screening. Prospective studies with landmark-based designs, larger at-risk subgroups, and robust regression methods such as Firth’s penalized Cox regression are needed before such clinical recommendations can be made with confidence.

## Figures and Tables

**Table 1:** Baseline (2019) characteristics of the study participants for each physical performance measure, stratified by risk status.

Measure	Variable	At Risk	No Risk	*p*-Value
Handgrip strength (HGS) (*n* = 226)				
	*n*	33	193	
	Age, years	80.242 ± 5.602	76.368 ± 5.484	<0.001
	BMI, kg/m^2^	23.270 ± 3.201	23.099 ± 4.347	0.829
	HGS, kg	16.167 ± 1.242	23.265 ± 3.252	<0.001
Timed Up and Go (TUG) (*n* = 225)				
	*n*	5	220	
	Age, years	83.400 ± 4.669	76.827 ± 5.592	0.010
	BMI, kg/m^2^	26.980 ± 2.742	23.050 ± 4.191	0.038
	TUG, s	13.304 ± 2.046	6.520 ± 1.151	<0.001
30-second chair stand test (CS-30) (*n* = 223)				
	*n*	18	205	
	Age, years	80.944 ± 6.485	76.580 ± 5.419	0.001
	BMI, kg/m^2^	23.524 ± 3.615	23.106 ± 4.265	0.687
	CS-30, repetitions/30 s	12.611 ± 2.279	23.302 ± 5.181	<0.001
One-leg standing test (OLST) (*n* = 226)				
	*n*	84	142	
	Age, years	79.845 ± 5.159	75.218 ± 5.239	<0.001
	BMI, kg/m^2^	24.051 ± 4.677	22.576 ± 3.795	0.010
	OLST, s	7.665 ± 4.123	46.638 ± 16.414	<0.001

Values are presented as mean ± standard deviation. BMI, body mass index; HGS, handgrip strength; TUG, Timed Up and Go test; CS-30, 30-second chair stand test; OLST, one-leg standing test. Between-group differences were assessed using the independent Student *t*-test. Shapiro–Wilk tests indicated that these variables were not normally distributed within the compared subgroups (*p* < 0.05 in all cases); a non-parametric sensitivity check (Mann–Whitney U test) yielded the same statistical conclusion (significant vs. non-significant at α = 0.05) as the *t*-test for 10 of the 12 baseline comparisons, with the exceptions being BMI for HGS and BMI for TUG (see Limitations Section).

**Table 2:** Characteristics of the study participants at the 4-year follow-up (2023) for each physical performance measure, stratified by risk status.

Measure	Variable	At Risk	No Risk	*p*-Value
Handgrip strength (HGS) (*n* = 117)				
	*n*	12	105	
	Age, years	83.333 ± 5.532	80.038 ± 5.179	0.040
	BMI, kg/m^2^	22.871 ± 2.897	23.621 ± 3.462	0.493
	HGS, kg	14.882 ± 2.643	22.542 ± 3.361	<0.001
Timed Up and Go (TUG) (*n* = 132)				
	*n*	11	121	
	Age, years	86.000 ± 5.477	80.529 ± 5.418	0.002
	BMI, kg/m^2^	23.177 ± 2.148	23.543 ± 3.368	0.726
	TUG, s	13.524 ± 1.973	7.110 ± 1.317	<0.001
30-s chair stand test (CS-30) (*n* = 124)				
	*n*	21	103	
	Age, years	84.333 ± 5.151	79.913 ± 5.189	<0.001
	BMI, kg/m^2^	23.608 ± 2.882	23.627 ± 3.374	0.981
	CS-30, repetitions/30 s	12.476 ± 2.182	22.806 ± 5.221	<0.001
One-leg standing test (OLST) (*n* = 84)				
	*n*	23	61	
	Age, years	81.957 ± 5.966	78.230 ± 4.466	0.003
	BMI, kg/m^2^	23.685 ± 4.254	23.685 ± 2.866	0.830
	OLST, s	7.826 ± 3.186	45.807 ± 15.756	<0.001

Values are presented as mean ± standard deviation. BMI, body mass index; HGS, handgrip strength; TUG, Timed Up and Go test; CS-30, 30-second chair stand test; OLST, one-leg standing test. Between-group differences were assessed using the independent Student *t*-test. Shapiro-Wilk tests indicated that these variables were not normally distributed within the compared subgroups (*p* < 0.05 in all cases); a non-parametric sensitivity check (Mann-Whitney U test) yielded the same statistical conclusion (significant vs. non-significant at α = 0.05) as the *t*-test for 10 of the 12 baseline comparisons, with the exceptions being BMI for HGS and BMI for TUG (see Limitations Section).

**Table 3:** Association between baseline (2019) physical performance and support/care-needs certification: Cox proportional hazards regression (Analysis 1).

Measure	Crude Model		Model 1 ^1^	
	HR (95% CI)	*p*-Value	HR (95% CI)	*p*-Value
HGS	2.19 (1.02–4.70)	0.044	1.21 (0.540–2.70)	0.646
TUG	3.29 (0.790–13.7)	0.102	1.66 (0.380–7.26)	0.501
CS-30	1.57 (0.553–4.46)	0.396	0.791 (0.265–2.36)	0.674
OLST	2.78 (1.42–5.48)	0.003	1.64 (0.792–3.40)	0.183

^1^ Model 1 was adjusted for age and body mass index. Certification events (n/N, %) by baseline risk status were: HGS, at risk 9/33 (27.3%), no risk 25/193 (13.0%); TUG, at risk 2/5 (40.0%), no risk 33/220 (15.0%); CS-30, at risk 4/18 (22.2%), no risk 30/205 (14.6%); OLST, at risk 21/84 (25.0%), no risk 14/142 (9.9%). Median follow-up was 4.0 years (i.e., the full study period) in all subgroups. HR, hazard ratio; CI, confidence interval; HGS, handgrip strength; TUG, Timed Up and Go test; CS-30, 30-second chair stand test; OLST, one-leg standing test.

**Table 4:** Association between 4-year (2023) physical performance and support/care-needs certification among participants without baseline risk: Cox proportional hazards regression (Analysis 2).

Measure	Crude Model		Model 1 ^1^	
	HR (95% CI)	*p*-Value	HR (95% CI)	*p*-Value
HGS	0.0420 (0.000–844)	0.531	0.00 (0–Inf)	0.985
TUG	29.7 (9.73–90.4)	<0.001	17.3 (4.86–61.8)	<0.001
CS-30	12.7 (3.91–41.4)	<0.001	7.09 (2.01–25.0)	0.002
OLST	8.07 (0.839–77.6)	0.071	5.12 (0.420–62.3)	0.201

^1^ Model 1 was adjusted for age and body mass index. This analysis targeted individuals classified as “no risk” at baseline. The at-risk subgroups in Analysis 2 are small (HGS, *n* = 12; TUG, *n* = 11; CS-30, *n* = 21; OLST, *n* = 23), which limits the precision and stability of the corresponding hazard ratio estimates; the HGS estimate in particular could not be reliably computed (see Section 3 and Section 4). Certification events (n/N, %) by 2023 risk status were: HGS, at risk 0/12 (0%), no risk 8/105 (7.6%); TUG, at risk 9/11 (81.8%), no risk 5/121 (4.1%); CS-30, at risk 9/21 (42.9%), no risk 4/103 (3.9%); OLST, at risk 3/23 (13.0%), no risk 1/61 (1.6%). Median follow-up was 4.0 years in all subgroups except the TUG at-risk group (median 3.0 years, reflecting its high event rate). HR, hazard ratio; CI, confidence interval; HGS, handgrip strength; TUG, Timed Up and Go test; CS-30, 30-second chair stand test; OLST, one-leg standing test.

**Table 5:** Sensitivity analysis excluding participants who had already been certified for Support/Care-Needs before their 2023 physical performance measurement: Cox proportional hazards regression (Analysis 2).

Measure	Crude Model		Model 1 ^1^	
	HR (95% CI)	*p*-Value	HR (95% CI)	*p*-Value
HGS	0.0430 (0.000–Inf)	0.839	0.00 (0.000–Inf)	0.997
TUG	39.0 (2.44–624)	0.010	19.0 (1.16–312)	0.039
CS-30	23,700 (0.000–1.78 × 10^39^)	0.806	1,090,000 (0.000–7.12 × 10^216^)	0.955
OLST	335 (0.000–1.80 × 10^9^)	0.462	42,200 (0.000–7.95 × 10^162^)	0.954

^1^ Model 1 was adjusted for age and body mass index. This analysis targeted individuals classified as “no risk” at baseline and additionally excluded participants who had received Support/Care-Needs certification before their 2023 re-measurement (HGS, *n* = 7 excluded; TUG, *n* = 12; CS-30, *n* = 11; OLST, *n* = 2), in order to evaluate the temporal-ordering concern that certification may have preceded, rather than followed, the assessed functional decline. For CS-30, OLST, and HGS, the hazard ratio could not be reliably estimated in this reduced sample (monotone likelihood; see Limitations Section), and the reported values should not be interpreted as stable effect estimates. The TUG association remained nominally significant but was based on very few remaining events and an extremely wide confidence interval. HR, hazard ratio; CI, confidence interval; HGS, handgrip strength; TUG, Timed Up and Go test; CS-30, 30-second chair stand test; OLST, one-leg standing test.

**Table 6:** Association between 4-year (2023) standardized continuous change (Δ) in physical performance and Support/Care-Needs certification: Cox proportional hazards regression using per-1-SD adverse change (Analysis 2).

Measure	Crude Model		Model 1 ^1^	
	HR (95% CI)	*p*-Value	HR (95% CI)	*p*-Value
HGS	1.06 (0.533–2.13)	0.858	0.677 (0.311–1.47)	0.325
TUG	2.40 (1.79–3.22)	<0.001	2.34 (1.64–3.33)	<0.001
CS-30	1.84 (1.11–3.07)	0.019	1.51 (0.890–2.57)	0.126
OLST	1.70 (0.548–5.24)	0.360	0.975 (0.248–3.83)	0.971

^1^ Model 1 was adjusted for age and body mass index. This analysis targeted individuals classified as “no risk” at baseline and used the standardized (z-scored) change in each measure from 2019 to 2023 (ΔHGS, ΔTUG, ΔCS-30, ΔOLST) as a continuous covariate, oriented so that a higher value represents functional worsening, in place of the binary “at-risk” classification used in Table 4. This analysis complements, but does not resolve, the temporal-ordering concern addressed by the exclusion-based sensitivity analysis in Table 5 (see Limitations Section). HR, hazard ratio; CI, confidence interval; SD, standard deviation; HGS, handgrip strength; TUG, Timed Up and Go test; CS-30, 30-second chair stand test; OLST, one-leg standing test.

## Data Availability

The data presented in this study are not publicly available because they contain information that could identify individual participants. During the ethical review process, it was explicitly agreed with participants that such identifiable data would not be disclosed publicly, in accordance with the approved study protocol (The Ethics Committee of the Prefectural University of Hiroshima, protocol code 24MH013).

## Supplementary Materials

The following supporting information can be downloaded at: https://www.mdpi.com/article/10.3390/healthcare14183009/s1, Table S1: Association between baseline (2019) physical performance and support/care-needs certification after multiple imputation (MICE) for missing baseline age and body mass index: Cox proportional hazards regression (Analysis 1); Table S2: Worst-case sensitivity analysis assigning participants who were unable to complete a given physical performance test to the “at-risk” category: association between baseline (2019) physical performance and support/care-needs certification; Cox proportional hazards regression (Analysis 1).

## Abbreviations

The following abbreviations are used in this manuscript:

ADL	Activities of daily living
AWGS	Asian Working Group for Sarcopenia
BMI	Body mass index
CI	Confidence interval
CS-30	30-second chair stand test
HGS	Handgrip strength
HR	Hazard ratio
JAGES	Japan Gerontological Evaluation Study
LTCI	Long-term care insurance
MADS	Musculoskeletal ambulation disability symptom complex
OLST	One-leg standing test
PPM	Physical performance measure
TUG	Timed Up and Go test
