# Supplementary material for: Impact of Longitudinal Changes in Dynamic Lower-Limb Function on Care-Needs Certification: A 4-Year Prospective Study of Older Women Attending Community Gathering Places (Kayoi-no-ba)
Source: Healthcare (Basel). 2026 Sep 14;14(18):3009. doi: 10.3390/healthcare14183009 (PMC13606191; doi:10.3390/healthcare14183009)
Supplement: Supplementary file 1 [file healthcare-14-03009-s001.zip › healthcare-4537304-supplementary.pdf]

Supplementary Materials

Impact of Longitudinal Changes in Dynamic Lower-Limb Function on Care-Needs Certification: A 4-Year Prospective Study of Older Women Attending Community Gathering Places (*Kayoi-no-ba*)

Table S1. Association between baseline (2019) physical performance and support/care-needs certification after multiple imputation (MICE) for missing baseline age and body mass index: Cox proportional hazards regression (Analysis 1).

Measure	**Crude Model**		**Model 1**[1]	
	**HR (95% CI)**	***p*-Value**	**HR (95% CI)**	***p*-Value**
HGS	2.77 (1.62–4.74)	<0.001	1.64 (0.936–2.88)	0.083
TUG	1.12 (0.031–40.3)	0.934	1.08 (0.046–25.2)	0.950
CS-30	1.22 (0.108–13.8)	0.840	0.856 (0.110–6.68)	0.854
OLST	2.20 (1.14–4.23)	0.021	1.29 (0.721–2.32)	0.377

1 Model 1 was adjusted for age and body mass index. Missing values for baseline age and body mass index (*n* = 150 of 608 eligible women) were addressed using multiple imputation by chained equations (Fully Conditional Specification method in IBM SPSS Statistics), generating 5 imputed datasets. Estimates were pooled across imputations using Rubin's rules. The imputation model included the physical performance measure under analysis (HGS, TUG, CS-30, or OLST risk category), baseline age, baseline BMI, the care-needs certification event indicator, and follow-up time as predictors. Pooled estimates were consistent with the complete-case analysis reported in Table 3 of the main text, and did not alter the study's conclusions. HR, hazard ratio; CI, confidence interval; HGS, handgrip strength; TUG, Timed Up and Go test; CS-30, 30-second chair stand test; OLST, one-leg standing test.

Table S2. Worst-case sensitivity analysis assigning participants who were unable to complete a given physical performance test to the “at-risk” category: association between baseline (2019) physical performance and support/care-needs certification, Cox proportional hazards regression (Analysis 1).

Measure	**Crude Model**		**Model 1**[1]	
	**HR (95% CI)**	***p*-Value**	**HR (95% CI)**	***p*-Value**
HGS	1.13 (0.686–1.87)	0.626	1.14 (0.656–1.99)	0.639
TUG	0.853 (0.522–1.39)	0.524	1.03 (0.597–1.78)	0.912
CS-30	0.907 (0.555–1.48)	0.696	0.960 (0.563–1.64)	0.882
OLST	1.68 (0.926–3.03)	0.088	1.37 (0.746–2.53)	0.308

1 Model 1 was adjusted for age and body mass index. Among the 458 women with baseline age and BMI available, participants who did not complete a given test (HGS, *n* = 232; TUG, *n* = 233; CS-30, *n* = 235; OLST, *n* = 232), for reasons that may include but are not limited to physical inability, were assigned to the “at-risk” category for that measure, representing the most conservative (worst-case) assumption about their true functional status. This differs from the complete-case analysis in Table 3 of the main text, which excludes these participants. Under this worst-case assumption, the adjusted (Model 1) associations remained non-significant for all four measures, consistent with the complete-case findings in Table 3, indicating that the exclusion of participants unable to complete testing did not materially bias the baseline (Analysis 1) results. HR, hazard ratio; CI, confidence interval; HGS, handgrip strength; TUG, Timed Up and Go test; CS-30, 30-second chair stand test; OLST, one-leg standing test.
